# The crucial factors influencing the development and outcomes of postoperative delirium in proximal femur fractures

**DOI:** 10.1002/agm2.12206

**Published:** 2022-04-26

**Authors:** Aysha Rajeev, Catherine Railton, Kailash Devalia

**Affiliations:** ^1^ Department of Trauma and Orthopaedics Gateshead Health Foundation NHS Trust Tyne and Wear UK

**Keywords:** delirium, development, outcomes, postoperative, proximal femur fractures

## Abstract

**Objective:**

The aim of this study is to find the incidence, risks, and reasons for prolonged length of hospital stay, short, long‐term mortality, and the factors contributing to mortality of postoperative delirium in proximal femoral fractures.

**Methods:**

The data for the study was obtained from National Hip Fracture Database (NHFD) and internal hospital computer systems (Medway, ICE, Clinic letters) between January 2018 and December 2019. One hundred seventy‐five patients were found have developed postoperative delirium. The outcomes measured were postoperative anemia, lower respiratory tract infection, urinary tract infection, acute kidney injury, urinary retention, cardiac event and stroke, alcohol or drug withdrawal, length of hospital stay, and 30 day and 1 year mortality.

**Results:**

The patients who developed delirium were 68 (38.9%) with American Society of Anesthesiologists (ASA) grade 4 and 94 (22.3%) without delirium (*p* < 0.05). The average length of stay after developing postoperative delirium was 19.69 days compared to 17.4 days for patients without delirium. The mortality at 30 days and 1 year was 10.9% and 37% in patients who had postoperative delirium compared to 2.1% and 2.8% to those without delirium, respectively.

**Conclusion:**

Postoperative delirium is three times more common in hip fractures. Early detection and timely management are crucial in the improvement of functional outcomes and mortality.

## Graphical abstract

Delirium has got serious implications in the morbidity, mortality, length of inpatient stay, and postoperative rehabilitation. The aim of this study is to find the incidence, identifiable causes, reasons for prolonged length of hospital stay, calculate short and long‐term mortality, and the factors contributing to mortality of postoperative delirium in proximal femoral fractures.

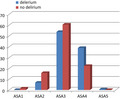

## 1 INTRODUCTION

The word delirium was first coined by Celsius in 1 AD and is derived from a Latin expression which means “off the track.”^1^ Delirium is defined as alteration in the mental status manifesting as acute onset of confusion, disorientation, changes in the sleep–wake rhythm, and level of consciousness. It is usually reversible.^2^ Delirium is common in elderly patients undergoing surgery for proximal femur fractures, the rates ranging from 4% to 53%.^3^ There are several factors that cause postoperative delirium and they are usually associated with poor outcomes. Delirium has got serious implications in the morbidity, mortality, length of inpatient stay, and postoperative rehabilitation and discharge destination in proximal femoral fractures.^4^, ^5^, ^6^

Pre‐existing mental illness and dementia in elderly patients is a risk factor in the development of postoperative delirium^6^ and sometimes predisposes to the development of dementia in hip fractures.^7^ The incidence of postoperative delirium is three times more common in hip fracture surgery compared with patients undergoing surgery for other indications.^8^ A better understanding of the underlying pathophysiology and the identifiable causes of postoperative delirium is vital in its prevention and management.^9^

The National Institute of Health and Clinical Excellence (NICE), in 2010, have focused on the importance of diagnosis, prevention, and management of postoperative delirium in patients with hip fractures.^10^ The current Best Practice Tariff (BPT) by NHS England has included a delirium assessment using the 4AT screening tool during the admission as one of the criteria.^11^ A multidisciplinary approach to identify those at risk and effective management of delirium in patients with hip fractures has been highlighted in the study by Chuan et al in 2020.^12^

The aim of this study is to find the incidence, identifiable causes, reasons for prolonged length of hospital stay, calculate short and long‐term mortality, and the factors contributing to mortality of postoperative delirium in patients with proximal femoral fractures.

## 2 METHODS

The data for the study was obtained from National Hip Fracture Database (NHFD) and internal hospital computer systems (Medway, ICE, Clinic letters). The study period was between January 2018 and December 2019. A total of 598 patients were admitted during the study period. The study was approved by the audit department of our hospital trust. This study used data from a public database and did not require ethical approval and patient consent.

**FIGURE 1:**
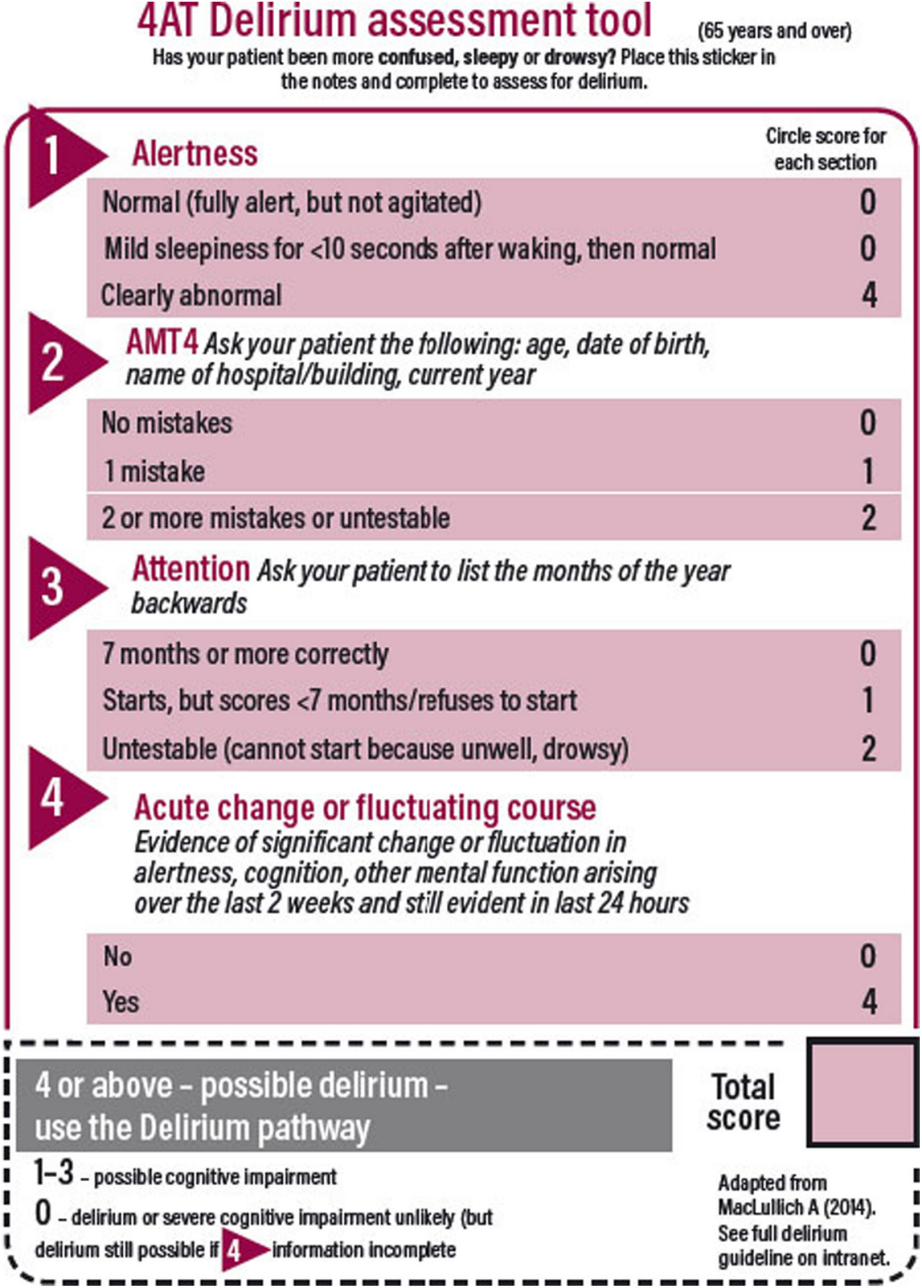
The 4AT assessment tool for delirium

After screening, 175 patients were found have developed postoperative delirium. The diagnosis of delirium was made using the 4AT scoring system (Figure 1). Patients aged 65 years or above who sustained a proximal femur fracture and underwent surgery and scored ≥4 in the 4AT assessment were included in the study. Patients with pathological fractures were excluded from the study.

All the patients admitted with proximal femur fracture were prospectively included in the database. Patient demographics including age, sex, fracture pattern, American Society of Anesthesiologists (ASA) grade, Abbreviated Mental Test (AMT) score at admission, type of anesthesia and procedure, comorbidities, complications, and mortality at 30 days and 1 year were recorded. Patients who had an AMT score of ≤6 were considered as having dementia. The comorbidities looked at are cardiovascular disease, mental illness, central nervous system disorders, chronic obstructive pulmonary disease (COPD), chronic kidney disease, malignancy, previous hip fractures, inflammatory arthritis, Parkinson’s disease, and chronic liver disease.

All patients were treated operatively with a postoperative protocol consisting of early mobilization with weight bearing. Patients were assessed by an experienced geriatrician pre and postoperatively. Delirium was identified from hospital chart notes and using 4AT delirium assessment criteria which included alertness, 4AMT, attention and change in cognitive or mental function, a score of ≥4 is considered as possible delirium.

The outcomes measured were postoperative anemia, lower respiratory tract infection, urinary tract infection, acute kidney injury, urinary retention, cardiac event, and stroke, alcohol or drug withdrawal, length of hospital stay, and 30 day and 1 year mortality. The effect of postoperative delirium on each of these outcomes was evaluated.

### 2.1 Statistical analysis

The statistical analysis was done using SPSS version 22. Comparison between patients who developed postoperative delirium and those without delirium were compared using chi‐squared, Student’s *t* test and Mann–Whitney *U* test for categorical, and parametric and nonparametric data sets, respectively. Logistic regression analysis was used to find the causes for postoperative delirium and also determining the factors contributing to mortality associated with delirium. A *p* value of <0.05 was considered significant.

## 3 RESULTS

**FIGURE 2:**
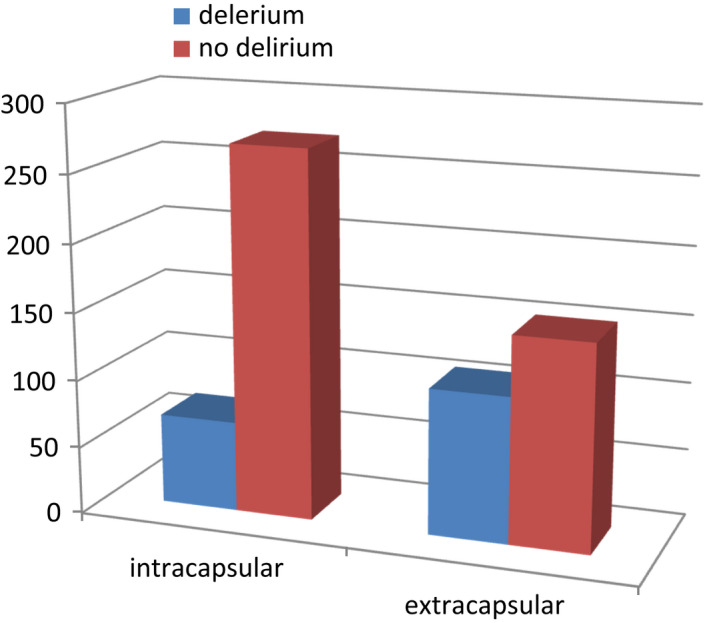
The proximal femur fracture pattern of patients with delirium and no delirium

The total number of patients during the study period admitted with proximal femur fractures was 598, after applying the inclusion and exclusion criteria. One hundred seventy‐five patients (29.3%) scored a 4AT of four or more, which suggests possible postoperative delirium. There were 68 (38.9%) men and 107 (61.1%) women in the delirium group and 124 (29.3%) men and 299 (70.7%) women in the group without delirium. The mean age for patients with delirium was 84.82 years (67–100 years) compared to 81.23 years in patients without delirium (*p* < 0.05). There were 67 patients (38%) with intracapsular fractures and 108 (62%) had extracapsular fractures, and in patients without delirium there were 271 (64.1%) intracapsular fractures and 152 (35.9%) extracapsular fractures (Figure 2).

**FIGURE 3:**
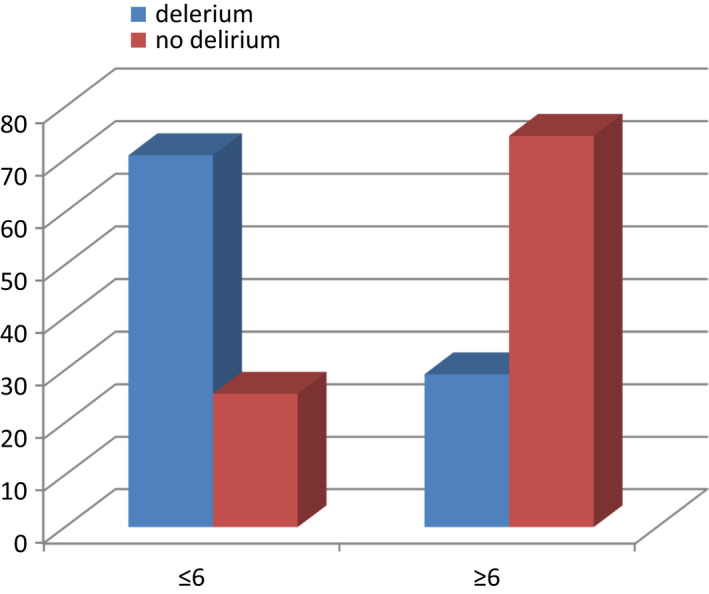
Abbreviated Mental Test (AMT) score of patients who had delirium and no delirium

**FIGURE 4:**
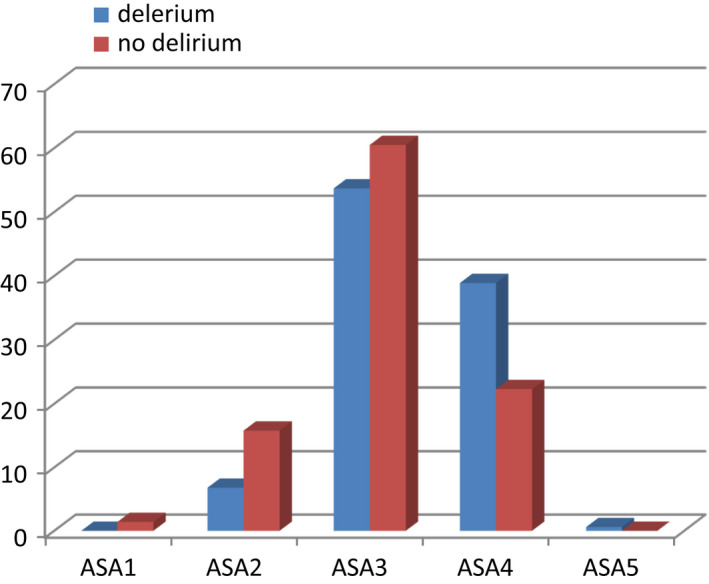
American Society of Anesthesiologists (ASA) grading of patients who had delirium and no delirium

A total of 124 patients (70.8%) had dementia (patients who scored ≤6 on the AMT score) compared to 108 (25.5%) patients without delirium (*p* < 0.05; Figure 3). The patients who developed delirium there were 68 (38.9%) with ASA grade 4 and 94 (22.3%) without delirium (*p* < 0.05; Figure 4).

The number of patients with pre admission comorbidities for delirium and non‐delirium were cardiovascular disease 125 (71.4%), 277 (65.5%), mental illness 102 (58.3%), 66 (15.6%), cerebrovascular disease including Parkinson’s disease 55 (31.4%), 102 (24.1%), COPD 49 (28%), 128 (30.2%), chronic kidney disease 33 (18.6%), 88 (21%), systemic malignancy 26 (14.9%), 26 (6.1%), previous hip fractures 13 (7.5%), 25 (5.9%), inflammatory arthritis 7 (4%), 21 (5%) and chronic liver disease 3 (1.7%), 8 (1.8%), respectively.

There were no significant statistical differences between patients who had general anesthesia and spinal anesthesia or the type of procedure carried out for the hip fractures in the delirium group and in the group without delirium. The baseline characteristics of patients in both groups are summarized in Table 1.

**TABLE 1:** Baseline patient characteristics

Patient variables	Delirium (%)	Without delirium (%)
No. of patients	175 (29.3)	423 (70.7%)
Age, mean ± SD	84.82 ± 16.84	81.23 ± 17.68
Sex		
Male	68 (38.9%)	124 (29.3%)
Female	107 (61.1%)	299 (70.7%)
ASA grade		
1	0	6 (1.4%)
2	12 (6.8%)	67 (15.8%)
3	94 (53.7%)	256 (60.5%)
4	68 (38.9%)	94 (22.3%)
5	1 (0.6%)	0
AMT score		
<6	124 (70.8%)	108 (25.5%)
≥6	51 (29.2%)	315 (74.5%)
Comorbidities		
Cardiovascular disease	125 (71.4%)	277 (65.5%)
Mental illness	102 (58.3%)	66 (15.6%)
Cerebrovascular disease	55 (31.4%)	102 (24.1%)
COPD	49 (28%)	128 (30.2%)
Chronic kidney disease	33 (18.6%)	88 (21%)
Systemic malignancy	26 (14.9%)	26 (6.1%)
Previous hip fracture	13 (7.5%)	25 (5.9%)
Inflammatory arthritis	7 (4%)	21 (5%)
Chronic liver disease	3 (1.7%)	8 (1.8%)
Fracture pattern		
Intracapsular	67 (38%)	271 (64.1%)
Extracapsular	108 (62%)	152 (35.9%)
Type of anesthesia		
General	139 (79.4%)	322 (76.1%)
Spinal	36 (20.6%)	101 (23.9%)
Procedure done		
Hemiarthroplasty	97 (55.4%)	234 55.3%)
Dynamic hip screw	46 (26.3%)	82 (19.4%)
Intramedullary nail	30 (17.1%)	70 (16.5%)
Total hip replacement	2 (1.2%)	37 (8.8%)

Abbreviations: AMT, Abbreviated Mental Test; ASA, American Society of Anesthesiologists; COPD, chronic obstructive pulmonary disease.

In our study, the identifiable factors which caused postoperative delirium were age more than 80 years (odds ratio [OR] = 1.05, 95% confidence interval [CI] = −1.02 to 1.09, *p* < 0.001), women (OR = 0.96, 95% CI = −0.62 to 1.53, *p* < 0.001), AMT scores ≤6 (OR = 3.80, 95% CI = −2.36 to 6.12, *p* < 0.001), ASA grade 4 (OR = 1.57, 95% CI = −1.01 to 2.42, *p* = 0.024), mental illness (OR = 1.65, 95% CI = −0.98 to 1.48, *p* < 0.001), postoperative anemia (OR = 0.91, 95% CI = −0.70 to 1.18, *p* < 0.001), systemic malignancy (OR = 0.91, 95% CI = −0.50 to 1.64, *p* < 0.001), lower respiratory tract infection (OR = 1.21, 95% CI = −0.98 to 1.48, *p* = 0.044), urinary tract infection (OR = 1.89, 95% CI = −1.20 to 3.00, *p* < 0.001), acute kidney injury (OR = 0.99, 95% CI = −0.98 to 0.99, *p* = 0.028), and urinary retention (OR = 1.90, 95% CI = −1.10 to 3.15, *p* = 0.016; Table 2).

**TABLE 2:** Identifiable causes of postoperative delirium

Causes	OR	95% CI	*p* value
Age > 80 years	1.05	1.02–1.09	<0.001
Females	0.96	0.62–1.53	<0.001
AMT score ≤6	3.80	2.36–6.12	<0.001
ASA grade 4	1.57	1.01–2.42	0.024
Mental illness	1.65	0.98–1.48	<0.001
Postoperative anemia < 8 g/L	0.91	0.70–1.18	<0.001
Systemic malignancy	0.91	0.50–1.64	<0.001
Lower respiratory tract infection	1.21	0.98–1.48	0.044
Urinary tract infection	1.89	1.20–3.00	<0.001
Acute kidney injury	0.99	0.98–0.99	0.028
Urinary retention	1.90	1.10–3.15	0.016

Abbreviations: AMT, Abbreviated Mental Test; ASA, American Society of Anesthesiologists; CI, confidence interval; OR, odds ratio.

The average length of stay after developing postoperative delirium was 19.69 days compared to 17.4 days for patients without delirium. The reasons for more than 20 days of inpatient stay were lower respiratory infection (17.9%, *p* < 0.001), acute kidney injury (10.4%, *p* = 0.693), sepsis (6%, *p* = 0.797), mental illness (49.3%, *p* < 0.001), and no cause were found in 16.4% (*p* < 0.001; Table 3).

**TABLE 3:** Reasons for more than 20 days of inpatient hospital stay

Causes	No.	Percentage	*p* value
Lower respiratory tract infection	12	17.9	<0.001
Acute kidney injury	7	10.4	0.693
Sepsis	4	6	0.797
Mental illness	33	49.3	<0.001
No cause	11	16.4	<0.001

The mortality at 30 days and 1 year was 10.9% and 37% in patients who had postoperative delirium compared to 2.1% and 2.8% to those without delirium, respectively, which was statistically significant (Table 4). The factors leading to high mortality were mental illness (OR = 1.25, 95% CI = −0.83 to 2.31, *p* = 0.021), postoperative anemia (OR = 1.09, 95% CI = −0.79 to 2.25, *p* = 0.035), lower respiratory tract infection (OR = 1.32, 95% CI = −0.72 to 2.35, *p* = 0.028), urinary tract infection (OR = 2.69, 95% CI = −1.02 to 7.23, *p* = 0.031), acute kidney injury (OR = 1.09, 95% CI = −1.01 to 2.03, *p* = 0.024), cardiovascular disease (OR = 1.27, 95% CI = −0.83 to 1.96, *p* = 0.027), and general anesthesia (OR = 1.24, 95% CI = 0.72–2.01, *p* = 0.031; Table 5).

**TABLE 4:** Mortality for 30 day and 1 year for the two groups

Mortality	Delirium, *N* (%)	Without delirium, *N* (%)	Total, *N* (%)	*p* value
30 days	19 (10.9)	9 (2.1)	28 (4.7)	<0.001
1 year	65 (37)	12 (2.8)	77 (12.9%)	<0.001

**TABLE 5:** Factors leading to high mortality

Causes	OR	95% CI	*p* value
Postoperative anemia	1.09	0.79–2.25	0.021
Mental health	1.25	0.83–2.31	0.035
Cardiovascular disease	1.27	0.83–1.96	0.027
Lower respiratory tract infection	1.32	0.72–2.35	0.028
Urinary tract infection	2.69	1.02–7.23	0.031
Acute kidney injury	1.09	1.01–2.03	0.024
General anesthesia	1.24	0.72–2.01	0.031

Abbreviations: CI, confidence interval; OR, odds ratio.

## 4 DISCUSSION

Surgery for proximal femur fractures in elderly and frail patients can often lead to altered disturbance in their mental status. It is usually manifested as dementia, depression, and delirium.^13^ The reported incidence of postoperative delirium varies from 9.5% to 61.3%.^14^, ^15^, ^16^ In our study, patients with fractures of the hip the incidence of postoperative delirium was 29.3%. But in a study by Edelstein et al, they noted an incidence as low as 5.1%.^17^ The reason for such a wide variation in the range of incidence of postoperative delirium may be because of the different diagnostic criteria used to diagnose delirium, such as Diagnostic and Statistical Manual, Confusion Assessment Method, and Mini‐Mental Status Examination. We used 4AT criteria for the diagnosis of delirium in our study.

There is strong association of pre‐operative AMT score and the development of postoperative delirium. A one point increase of AMT score can lead to a fall in the odds of postoperative delirium by 0.6 fold.^18^ Yang et al in their meta‐analysis found that age and pre‐operative dementia to be a risk factor for the development of delirium.^19^ Another study by Kim et al also made similar observations.^20^ In the 2018 report by the NHFD, they suggested that an AMT score of less than 8 is a significant predictor of postoperative delirium in hip fractures using the 4AT diagnostic criteria.^21^ In our study, we used an AMT score ≤6 as a threshold for cognitive impairment. Of the patients, there were 70.8% with an AMT score ≤6 who went on to develop postoperative delirium in comparison with 25.5% with similar AMT scores who did not have delirium.

The functional reserve of the patients with hip fractures has been postulated as one of the most important factors leading to the development of postoperative delirium.^18^ This is indicated by the ASA grade. The risk of developing postoperative delirium increases by twofold if the ASA grade goes up one grade.^6^ In our study also, of the patients with ASA grade 4, 39% developed postoperative delirium. There are several studies which showed that the presence of cardiac and respiratory diseases predisposes to postoperative delirium.^19^, ^20^, ^22^ These findings were in contrast to our observation where no significant differences were noted between delirium and non‐delirium.

In the 2018 NHFD report, it was suggested that there is an increased incidence of postoperative delirium in patients who had general anesthesia for the surgical repair of hip fractures.^21^ Anh et al, in their retrospective study, found that by administering regional anesthesia for this cohort of patients the incidence of postoperative delirium was reduced by 2.5 times.^23^ But our study has demonstrated no difference between general and regional anesthesia in the development of delirium for hip fracture surgery. This is in full agreement to other published studies.^18^, ^24^, ^25^

The average age of our patients who developed delirium was 84.82 years. Advancing age is one of the risk factors of delirium.^26^, ^27^, ^28^ The elderly patients who sustain hip fractures are octogenarians and with multiple comorbidities.^29^ In the current study, pre‐existing cardiovascular disease, mental illness, and cerebrovascular diseases had a significant role in the development of postoperative delirium. The studies by Zakriya et al^30^ and Edlund et al^31^ also found the same observations for cardiovascular and mental diseases, respectively. Edelstein et al^17^ also observed the role of cerebrovascular disease in the development of delirium similar to our study.

Postoperative anemia in our study is one risk factor identified. There are a number of studies describing the incidence of low hemoglobin level to be associated with delirium and post blood transfusion to be associated with a lower delirium incidence.^32^, ^33^ Our study also showed increased incidence of delirium with lower respiratory and urinary tract infections with retention. But the study by Balogun and Philbrick^34^ has concluded that there is no definite association between urinary tract infection (UTI) and delirium. Kuswardhani and Sugi^35^ in their study have observed that sepsis in the nature of lower respiratory or urinary tract infections has got a strong relationship with the severity of delirium.

Delirium associated with increased incidence of postoperative complications including pneumonia, UTI, cardiovascular accident, and pulmonary complications in general leads to prolonged inpatient stay which in turn has got financial implications.^6^, ^36^ The average length of stay in our study was 2.24 days more for patients with delirium compared to that of non‐delirious patients. The previous studies by Arshi et al.^37^ and Tahir et al.^29^ also reported an average length of stay to about 3 days.

The meta‐analysis done by Hamilton et al.^38^ has identified postoperative delirium as a significant factor affecting the mortality. In the current study, the 30 day and 1 year mortality was 11% and 37%, respectively. This is significantly higher than the patients without delirium. We found that the factors associated with increased mortality in patients with delirium, such as postoperative anemia, lower respiratory tract infection, UTI, acute kidney injury, cardiovascular disease, and general anesthesia are modifiable and reversible with early diagnosis and treatment.

## 5 CONCLUSION

The development of postoperative delirium in hip fractures most often is associated with pre‐existing morbidities. In general, these factors can be identified and reversed by a multidisciplinary team approach. Our study highlights the risk factors in the perioperative period influencing the development and outcomes associated with postoperative delirium.

## 6 ACKNOWLEDGMENTS

The authors wish to acknowledge Dr. Mike Wilkinson, Associate Specialist in Elderly Medicine, Queen Elizabeth Hospital, Gateshead, UK for delirium data assimilation and profiling.

## 7 CONFLICT OF INTEREST

Nothing to disclose.

## 8 AUTHOR CONTRIBUTIONS

Aysha Rajeev contributed to study design, analysis and writing of the manuscript. Catherine Railton contributed to the data collection. Kailash Devalia contributed to the review of the manuscript.
